# Sirtuin-1 and Its Relevance in Vascular Calcification

**DOI:** 10.3390/ijms21051593

**Published:** 2020-02-26

**Authors:** Chien-Lin Lu, Min-Tser Liao, Yi-Chou Hou, Yu-Wei Fang, Cai-Mei Zheng, Wen-Chih Liu, Chia-Ter Chao, Kuo-Cheng Lu, Yee-Yung Ng

**Affiliations:** 1 Division of Nephrology, Department of Medicine, Fu Jen Catholic University Hospital, School of Medicine, Fu Jen Catholic University, New Taipei City 242, Taiwan; janlin0123@gmail.com (C.-L.L.); yyngscwu12@gmail.com (Y.-Y.N.); 2 Department of Pediatrics, Taoyuan Armed Forces General Hospital, Taoyuan City 325, Taiwan; liaoped804h@yahoo.com.tw; 3 Department of Pediatrics, Tri-Service General Hospital, National Defense Medical Center, Taipei 114, Taiwan; 4 School of Medicine, College of Medicine, Fu-Jen Catholic University, New Taipei City 24205, Taiwan; athletics910@gmail.com (Y.-C.H.); m005916@gmail.com (Y.-W.F.); 5 Division of Nephrology, Department of Medicine, Cardinal-Tien Hospital, School of Medicine, Fu-Jen Catholic University, New Taipei City 234, Taiwan; 6 Division of Nephrology, Department of Internal Medicine, Shin-Kong Wu Ho-Su Memorial Hospital, Taipei 111, Taiwan; 7 Graduate Institute of Clinical Medicine, College of Medicine, Taipei Medical University, Taipei 110, Taiwan; 11044@s.tmu.edu.tw (C.-M.Z.); wayneliu55@gmail.com (W.-C.L.); 8 Division of Nephrology, Department of Internal Medicine, School of Medicine, College of Medicine, Taipei Medical University, Taipei 110, Taiwan; 9 Division of Nephrology, Department of Internal Medicine, Shuang Ho Hospital, Taipei Medical University, Taipei 235, Taiwan; 10 Division of Nephrology, Department of Internal Medicine, Tungs’ Taichung MetroHarbor Hospital, Taichung City 433, Taiwan; 11 Graduate Institute of Toxicology, National Taiwan University College of Medicine, Taipei 104, Taiwan; 12 Nephrology division, Department of Internal Medicine, National Taiwan University Hospital, Taipei 100, Taiwan; 13 Department of Internal Medicine, National Taiwan University Hospital BeiHu Branch, Taipei 108, Taiwan

**Keywords:** sirtuin-1, vascular calcification, endothelial cells, vascular smooth muscle cells, perivascular adipose tissue

## Abstract

Vascular calcification (VC) is highly associated with cardiovascular disease and all-cause mortality in patients with chronic kidney disease. Dysregulation of endothelial cells and vascular smooth muscle cells (VSMCs) is related to VC. Sirtuin-1 (Sirt1) deacetylase encompasses a broad range of transcription factors that are linked to an extended lifespan. Sirt1 enhances endothelial NO synthase and upregulates FoxOs to activate its antioxidant properties and delay cell senescence. Sirt1 reverses osteogenic phenotypic transdifferentiation by influencing RUNX2 expression in VSMCs. Low Sirt1 hardly prevents acetylation by p300 and phosphorylation of β-catenin that, following the facilitation of β-catenin translocation, drives osteogenic phenotypic transdifferentiation. Hyperphosphatemia induces VC by osteogenic conversion, apoptosis, and senescence of VSMCs through the Pit-1 cotransporter, which can be retarded by the sirt1 activator resveratrol. Proinflammatory adipocytokines released from dysfunctional perivascular adipose tissue (PVAT) mediate medial calcification and arterial stiffness. Sirt1 ameliorates release of PVAT adipokines and increases adiponectin secretion, which interact with FoxO 1 against oxidative stress and inflammatory arterial insult. Conclusively, Sirt1 decelerates VC by means of influencing endothelial NO bioavailability, senescence of ECs and VSMCs, osteogenic phenotypic transdifferentiation, apoptosis of VSMCs, ECM deposition, and the inflammatory response of PVAT. Factors that aggravate VC include vitamin D deficiency-related macrophage recruitment and further inflammation responses. Supplementation with vitamin D to adequate levels is beneficial in improving PVAT macrophage infiltration and local inflammation, which further prevents VC.

## 1. Introduction

The presence of vascular calcification (VC) is highly associated with an increased risk of cardiovascular disease and all-cause mortality [1,2,3]. Patients with advanced age, diabetes mellitus (DM), and chronic kidney disease (CKD) are at risk for VC. Less commonly, vitamin D toxicity, vitamin K deficiency or antagonist, and osteoporosis are reported in relation to VC [4]. VC in small or medium-sized arteries is described as Monckeberg’s sclerosis, discovered by Johann Georg Monckeberg in 1903 [5], and characterizes progressive stiffening of the elastic layer in the arterial wall. The traditional theory to activate calcification says that high phosphate levels activate the osteogenic phenotype transition of VSMCs directly by the activation of renin–angiotensin–aldosterone system (RAAS)-related inflammatory responses [6]. Protein-bound uremic toxin can further aggravate this active calcification process in endothelial and medial layers of the vessel [7]. New concepts indicated that calciprotein particles (CPPs) and matrix vesicles (MVs) participate in passive calcification, which can be triggered by hyperphosphatemia and protein-bound uremic toxin-related inflammatory reactions, and lead to calcification within the medial layer of vessel [8,9,10]. In addition to the medial layer, VC can also occur in the intimal layer of the vessel wall. Intimal calcification is generally associated with atherosclerosis, which is an inflammatory dependent process and leads to plaque calcification. In atherosclerotic lesions, macrophages contribute to the maintenance of the local inflammatory response that facilitates calcification, and they release inflammatory cytokines such as interleukin (IL)-1β, IL-6, tumor necrosis factor-α (TNF-α), serpina3 and oncostatin M that promote VSMCs osteogenic differentiation and further mineralization [11,12,13,14]. In addition, local vessel wall inflammation could be accelerated by circulating immune cells adhering to de-amidated endothelial proteins that enhance the release of inflammatory cytokines and, thereby, the development of atherosclerotic plaque [15].

## 2. The Diverse Function of Sirtuin1 in Physiology and Clinical Disease

**Figure 1:**
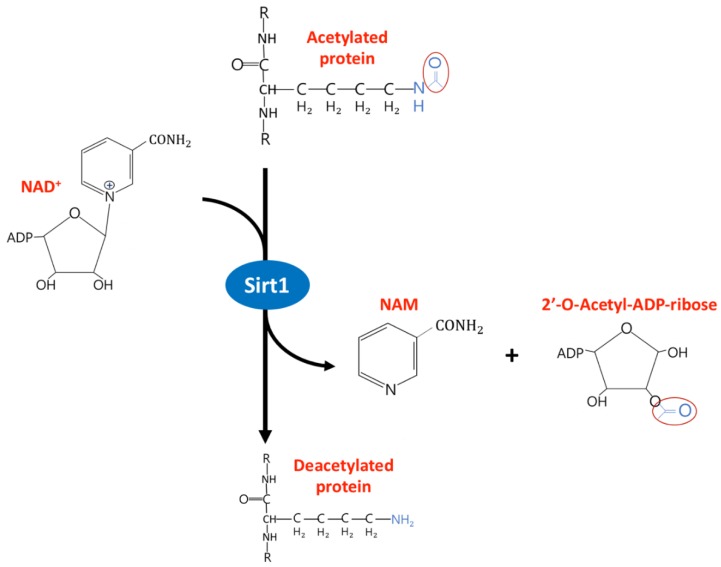
Enzyme activity of Sirtuins-1. NAD+ receive acetyl-lysine residues from the target protein during the deacetylation process and generate nicotinamide (NAM) and 2′-O-Acetyl-ADP-ribose. NAD, Nicotinamide adenine dinucleotide and NAM, nicotinamide.

The Sirtuin family has seven subtypes in mammals: sirtuin1 to 7. Sirtuins are categorized as class III lysine deacetylases by removing acetyl groups of lysine side chains of various substances with the help of coenzyme nicotinamide adenosine dinucleotide 1 (NAD1), and NAD1 is subsequently converted to nicotinamide (NAM) and 2′-O-Acetyl-ADP-ribose (Figure 1). Sirtuin1 (Sirt1) and Sirt6/7 are predominantly located in the nucleus, Sirt2 in the cytoplasm, and Sirt3/4/5 in the mitochondria. Sirtuin is implicated in influencing cellular hemostasis, including glucose/lipid metabolism, inflammation, oxidative stress, senescence, and cancer [16]. Sirtuin can directly deacetylate histones to regulate chromatin function and modulate histones and DNA methylation epigenetically [17,18]. Non-histone proteins, including the forkhead box-containing protein type O subfamily (FoxO), p53 transcription factor, nuclear factor-κB (NFκB), peroxisome proliferator activated receptor (PPAR), histone acetyltransferase (HAT) p300, superoxide dismutase (SOD), and adenine translocator (ANT), along with the metabolic enzymes acetyl-CoA synthetases (AceCSs), are the possible substances regulated by Sirtuin. For example, Sirt1 deacetylates the p53 protein, which attenuates its transcription activity in response to DNA damage and inhibits the p53-dependent apoptotic response [19,20]. 

Sirt1 is linked to extended lifespans and acts as an intracellular energy sensor involved in the pathophysiology of aging [21,22,23] and fatty or non-fatty liver disease. The NAD^+^/NADH ratio is a key regulator that determines Sirt1 activity. Alcohol, a high-fat diet, and/or a high-caloric diet impede Sirt1 activity by decreasing the NAD^+^/NADH ratio and results in the development of alcoholic fatty liver disease. For example, alcohol metabolism in hepatocytes convert acetaldehyde to acetate and protonate NAD+ to NADH [24,25]. Whereas, calorie restriction (CR) conversely increases the NAD^+^/NADH ratio and, subsequently, increases Sirt1 activity, which is why cumulative evidence supports CR as a possible way to enhance longevity in mammals [26]. 

Sirt1 regulates cell metabolism in various tissue by regulating a number of molecules, including PPARα, peroxisome proliferator-activated receptor-gamma co-activator 1 alpha (PGC-1α), and NF-κB. For instance, Sirt1 interacts with PPARα to deacetylase coactivator PGC-1α to protect cardiac hypertrophy and prevent fatty acid dysregulation in cardiomyocytes [27]. The relationship between Sirt1 and PPARγ is also identified in cellular senescence [28]. In hepatocytes, Sirt1 promotes gluconeogenesis to tolerate prolonged fasting by utilizing PGC-1α and FoxO1 to facilitate CREB-regulated transcription coactivator 2 (CRTC2) degradation [29]. Additionally, long-term fasting enhances keto body production as an energy source via the interaction between Sirt1 and PPARα activation and inhibits fatty acid synthesis through deacetylase sterol regulatory element binding protein 1c (SREBP1c) expression [30,31]. Sirt1 can act as a cholesterol sensor to regulate whole-body cholesterol and lipid hemostasis by positive regulation of liver X receptor (LXR) proteins [32]. In skeletal muscles, mitochondrial fatty acid oxidation is induced by Sirt1 deacetylation of PGC-1α, and this process is essential during food deprivation [33]. During glucose restriction, Sirt1 causes the deacetylation of glycolytic enzyme phosphoglycerate mutase-1 (PGAM1), negatively regulating glycolysis and diminishing energy production in skeletal muscles [34]. Clinically, Sirt1 has a protective role in preventing metabolic disease, cardiovascular disease, diabetes, neurodegenerative disease, and cancer [35]. 

## 3. The Protective Role of Sirt1 Against Vascular Calcification 

### 3.1. Sirt1 Regulate Nitric Oxide and eNOS Expression in Endothelium

**Figure 2:**
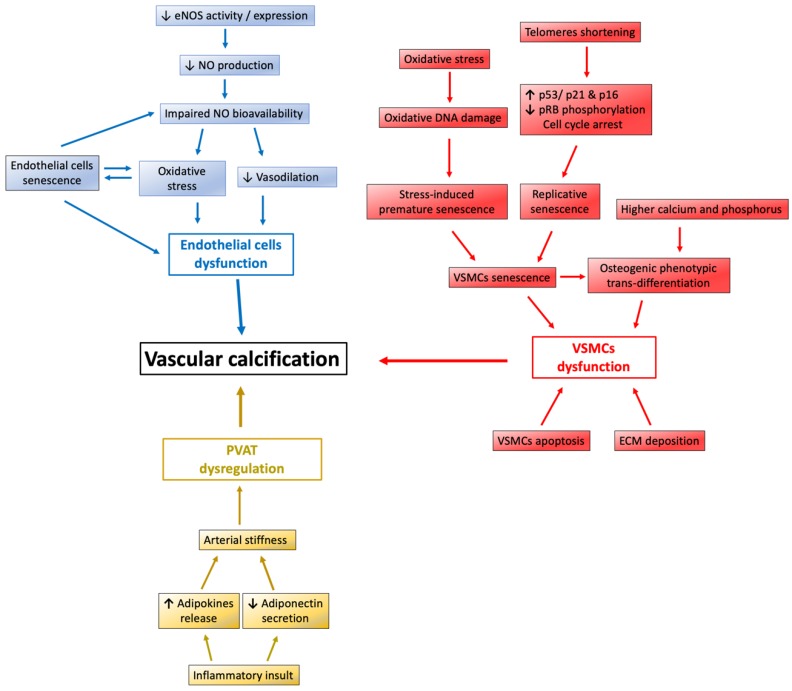
The factors contribute to the development of vascular calcification. In endothelium, endothelial-derived NO has vasodilation and antioxidant properties to protect against atherosclerosis and oxidative stress. Senescence of both ECs and VSMCs contributes to VC. Senescent VSMCs exhibit a transition into the osteoblastic phenotype, which plays a crucial role in developing vascular medial calcification. Higher calcium and higher phosphorus also lead to osteogenic phenotype transdifferentiation. PVAT has the ability to release proinflammatory adipokines and, consequently, promote VSMCs proliferation. ECM, extracellular matrix; NO, nitric oxide; NOS, NO synthase; PVAT, perivascular adipose tissue; and VSMCs, vascular smooth muscle cells.

Nitric oxide (NO) is synthesized in the endothelium from the conversion of L-arginine and is catalyzed by the calcium-calmodulin control enzyme endothelial NO synthase (eNOS). NO is a soluble gas that is synthesized within the cytosol of endothelial cells (ECs), and it diffuses rapidly into adjacent vascular smooth muscle cells (VSMCs). In VSMCs, NO exerts its vasodilation function via at least two mechanisms. NO activates guanylyl cyclase to increase the synthesis of 3,5-cyclic guanosine monophosphate (3,5-cGMP) and, consequently, leads to vasorelaxation without changing the intracellular calcium level; NO decreases the intracellular calcium concentration by calcium uptake into the endoplasmic reticulum, leading to inhibition of the calcium-calmodulin myosin light chain kinase complex in VSMCs and to vasorelaxation [36,37]. Moreover, NO can react with reactive oxygen species (ROS) and diminish its oxidative damage. ROS play a crucial role in the pathogenesis of cardiovascular disease [38]. Disturbance in the redox balance causes ROS production and oxidative stress, which is harmful for ECs and leads to endothelial dysfunction and senescence by telomere shortening [39,40]. Therefore, endothelial-derived NO has vasodilation and antioxidant properties to protect against atherosclerosis and oxidative stress (Figure 2, blue). A deficiency of endothelial NO bioavailability, whether vascular NO reduction or eNOS inhibition, is related to blood flow reduction in humans [41,42,43] or increase in vascular resistance leading to high blood pressure in animals [44,45,46]. Actually, endothelial NO is not the only mechanism for preventing inflammation and calcification of the vessel wall. It is important to note that every single cardiovascular event leads to transcriptional change in the vessel wall, especially VSMCs. In patients with acute myocardial infarction, aortic VSMCs transcript alteration leads to upregulation of the promyogenic modulation of Myoc and Muscleblind-like splicing regulator 1 (MBNL1) genes that influence VSMC proliferation and differentiation [47,48,49].

Sirt1 has the ability to enhance eNOS deacetylation and promote its activity, which increases NO production in ECs and leads to endothelial-dependent vasodilation [50]. Sirt1 activator can upregulate eNOS transcription and activity to increase eNOS and then NO production in epithelial cells [51]. Conversely, inhibiting Sirt1 by interference RNA decreases NO production and inhibits vasodilation. Double immunofluorescence assay revealed Sirt1 and eNOS are colocalized in the perinuclear cytoplasm of ECs. Therefore, these findings suggest Sirt1 has a direct role in modulating vasodilation through the expression of eNOS. Surprisingly, eNOS can conversely regulate Sirt1 expression in ECs. Ota et al. demonstrated that cilostzole increase eNOS activity against oxidative stress in ECs by phosphorylation of eNOS at Ser^1177^ and the subsequent increase in Sirt1 expression [52]. In ECs, the interaction between Sirt1 and eNOS/NO is interesting, because they can regulate each other synergistically in a positive feedback autoloop.

### 3.2. Sirt1 Affect Endothelial Cells and VSMCs Function against Vascular Calcification

#### 3.2.1. Sirt1 Abolish Endothelial Cells and VSMCs Senescence 

Senescence of both ECs and VSMCs contributes to VC and is implicated in the process of cardiovascular disease. In a senescent cell, the morphology and gene expression pattern are altered and impair cell function, which enhances the risk of vascular aging, inflammation, and calcification. eNOS activation has the benefit of delaying endothelial cellular senescence [40]. However, in senescent ECs, eNOS expression is downregulated and, consequently, susceptible to oxidative stress injury. CR is a dietary regimen that induces eNOS expression and is accompanied by enhanced Sirt1 levels, which is considered a link to delay aging and extend the lifespan in mammals [53]. The Forkhead box O (FoxO) transcription factor is an important element to maintain endothelial morphology [54], inducing EC apoptosis [55] and inhibiting angiogenesis [56,57,58], which have protective roles against oxidative injury. FoxO activity is mainly regulated by protein kinase B (Akt), which directly phosphorylates FoxO factors and leads to nuclear/cytoplasmic shuttling of FoxOs [59,60]. FoxO’s transcription factors are downstream signals of Sirt1, and activation of Sirt1 induces FoxO3a expression to suppresses cellular ROS production under a hyperglycemic environment [61]. In addition, Sirt1 overexpression prevents oxidative stress-induced apoptosis through FoxO3a inhibition in endothelial progenitor cells (EPCs), which function to repair vascular injury [62]. This accumulative evidence supports that Sirt1/FoxO has a direct role in oxidative stress resistance in ECs and are a potential target to treat related vascular diseases.

VSMCs senescence contributes to aging and age-related disease [63]. The cause of VSMCs senescence can be categorized into two different processes: replicative senescence in plaque and stress-induced premature senescence (SIPS) by oxidative stress (Figure 2, red). Both types of senescence injury lead to atherosclerosis plaque instability and VSMCs proliferation in impairing the repair process, which predisposes one to VC and cardiovascular disease. Telomeres consist of long stretches of TTAGGG DNA repeats, and they play a protective role by recognizing DNA double-stranded breaks and activating the DNA damage response mechanism [64]. During somatic cell division, telomeres are progressively shortened and finally result in irreversible replicative senescence. Replicative senescence is associated with increased expression of p16 and p21 proteins, which are responsible for CKD inhibition and, subsequently, decreased retinoblastoma protein (RB) phosphorylation. By inhibiting the activity of the E2F transcription factor, hypophosphorylated RB blocks the transition of the cell cycle from the G1 phase into the S phase [65,66,67]. SIPS is characterized by increasing the oxidative stress burden and inducing global oxidative DNA damage and irreversible cell growth arrest in VSMCs. Since angiotensin II (Ang II) plays a central role in the pathogenesis of cardiovascular disease and VSMCs is the target of Ang II action [68,69,70], Ang II stimulates the production of ROS in VSMCs. This mediates DNA damage and fragmentation, especially H_2_O_2_, to undergo acute SIPS and replicative senescence with consequent accelerated telomere attrition [69]. Additionally, the protein level of Sirt1 in human VSMCs decreases as age increases. Age-related loss of Sirt1 expression in human VSMCs leads to several cell functional deficits, such as impaired stress response to UVB, reduced capacity for VSMCs migration and proliferation, and induction of cellular senescence [71]. Resveratrol, a Sirt1 activator, can attenuate DNA damage following exposure to UVB stress inducers. 

#### 3.2.2. Sirt1 Attenuates the Osteoblastic Phenotypic Transition of VSMCs 

In general, smooth muscle cells are characterized by slowly proliferative nonvoluntary contractile cells, found in a variety of tissues. In blood vessels, contractile type smooth muscle expresses several contraction-related proteins, such as SM α-actin (SMαA), SM-22α, SM myosin heavy chains SM-1 and SM-2, calponin, and smoothelin, to regulate blood pressure and maintain the extracellular matrix of blood vessels. However, smooth muscles cells are not terminally differentiated cells and have phenotypic plasticity. In response to local insults, like injury or inflammation, smooth muscle cells are predisposed to enter a synthetic state by downregulation of contractile proteins, increasing the extracellular matrix to facilitate migration by enhancing its migration ability. The transition from contractile phenotype to osteoblastic phenotype is characterized by the release of calcifying vesicles, loss of the marker of contraction-related proteins, and gain of osteoblastic-like proteins, such as runt-related transcription factor-2 (Runx2), osteopontin, osteocalcin, alkaline phosphatase (ALP), and Type I collagen [72,73].

Senescent cells have impaired cellular division and regenerative capacity, and they are considered as a different loss-of-function cell type [74]. Several molecule changes have been studied in senescent cells, such as β-galactosidase activity, p53, p21, phosphorylated H2A histone family member X (γH2AX), and p38 mitogen-activated protein kinase (p38MAPK), which reflect activation of DNA damage [75,76]. Senescence drives the phenotypic switch of VSMCs toward an osteoblast-like phenotype [77] and is characteristic with osteoblastic phenotype by higher levels of ALP and type I collagen, which plays a crucial role in developing vascular medial calcification. Moreover, RUNX2 is upregulated in senescent VSMCs and displays prominent medial calcification. 

**Figure 3:**
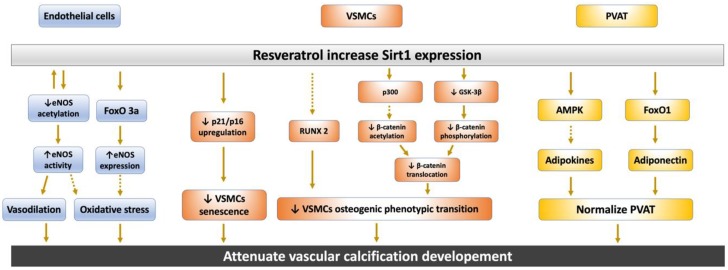
The protective role of Sirt1 activator resveratrol in attenuating vascular calcification. In epithelial cells, Sirt1 and eNOS/NO can regulate each other in a positive feedback loop to possess significant antioxidant and anti-inflammatory properties. Sirt1 inhibit the upregulation of the p16 and p21 expression and thereby delay cell VSMCs senescence. Additionally, Sirt1 directly inhibit osteogenic phenotypic transdifferentiation of VSMCs through deacetylation of RUNX2 and β-catenin. Sirt1 protects against oxidative stress and inflammatory insult by preventing adipocytokine release through the AMPK pathway or normalizing adiponectin secretion in PVAT. AMPK, adenosine monophosphate-activated protein kinase; FoxO, Forkhead box O; GSK-3β, glycogen synthase kinase-3 beta; NO, nitric oxide; NOS, NO synthase; RUNX2, runt-related transcription factor-2; PVAT, perivascular adipose tissue; and VSMCs, vascular smooth muscle cells. (Solid arrow: promote; Dotted arrow: inhibit)

It is well-known that high dosages of vitamin D promote VC. Administration of pharmacological doses of vitamin D in experimental animals caused widespread arterial wall calcification. Vitamin D toxicity in humans is associated with extensive arterial calcium phosphate deposition. Vitamin D induces VC by several mechanisms, including increasing serum calcium and phosphate, formation of fetuin-A mineral complex and a decrease of free fetuin-A levels, and inducing osteoblastic phenotypic transition of VSMCs [78]. VSMC exposure to hyperglycemia can further accelerate the senescent process with a higher degree of mineralized matrix deposition and induce β-galactosidase activity, upregulating the cell cycle markers p16 and p21. Additionally, Sirt1 activator can alleviate VSMC mineralization by upregulating the expression of the calcification inhibitors OPG and OPN. More importantly, Sirt1 directly acts on the RUNX2 level in hyperglycemic conditions by deacetylation of the RUNX2 promoter (Figure 3). Consequently, Sirt1 possesses the characteristic of anti-calcification and plays a key role in perpetuating VC [79]. By the way, microRNA-34a promotes VC by downregulating AXL receptor tyrosine kinase (Axl) and Sirt1, resulting in VSMCs senescence and mineralization [80]. 

The myocardin (MyoC) gene is a myogenic coactivator expressed mainly in cardiac and smooth muscle cells that is responsible for contractile phenotype of VSMC transition. According to the functional interaction map, the Sirt1 gene directly interacts with the MyoC gene. Downregulation of the contractile phenotype of VSMCs propagates inflammatory macrophages and VSMC senescence. Maintaining the normal contractile phenotype of VSMCs is considered a protective mechanism against vessel wall inflammation and further VC [81]. 

### 3.3. Sirt1 Restore Perivascular Adipose Tissue Dysregulation

Perivascular adipose tissue (PVAT) is adipose tissue that surrounds large arteries and releases various factors, in paracrine or autocrine fashion, in the regulation of vascular function. PVAT has the ability to release proinflammatory adipokines or cytokines after insult and, consequently, promote inflammation, vasoconstriction, and VSMCs proliferation, which are all highly associated with cardiovascular risk, especially in obesity (Figure 2, yellow) [82]. Numerous studies have shown that PVAT plays a protective role against atherosclerosis by increasing the adiponectin level and eNOS expression, thereby inhibiting plaque formation, reducing inflammation, and improving endothelial function [83]. Furthermore, these PVAT-derived factors are capable of developing arterial stiffness and are thought to be associated with VC [84]. Clinically, thoracic PVAT mass is correlated with coronary artery calcification in HIV-infected patients [85]. Additionally, in systemic lupus erythematosus patients, median thoracic aortic PVAT and aortic calcification are positively correlated [86]. 

The activation of Sirt1 can abolish dysregulated PVAT adipokine release after inflammatory insult [87] and also reduce inflammatory cytokines release to improve arterial wall hypertrophy and adventitial collagen I accumulation, which resulted in arterial stiffness in aged mice [88]. Sirt1 in PVAT is also proven to regulate adiponectin secretion through the interaction with FoxO1 protein [89]. Adiponectin is an adipocyte-derived protein that is important in regulating carbohydrate metabolism, and it also possesses anti-atherosclerotic characteristics on the vascular endothelium [90]. To sum up, Sirt1 protects against oxidative stress and inflammatory insult by preventing adipocytokine release through the adenosine monophosphate-activated protein kinase (AMPK) pathway or normalizing adiponectin secretion in PVAT [91].

## 4. The Interplay between Sirt1 and the Wnt/β-Catenin Pathway in Vascular Calcification

Wnt signaling has been shown to play a crucial role in bone development, and the Wnt ligand can tightly control the coupling of bone formation and bone resorption by modulating osteoblast and osteoclast differentiation from mesenchymal progenitor cells. Canonical Wnt signaling is activated, since the Wnt ligand binds to a dual receptor complex comprising the Wnt co-receptor LRP 5/6 and one of the seven transmembrane receptors of the FZD family. Subsequently, dephosphorylated β-catenin has the ability to translocate into the nucleus where it binds to T cell factor/lymphoid enhancer factor (TCF/LEF) transcription factors and then initiates transcription of various genes. 

Both canonical and noncanonical Wnt-signaling pathways are involved in the phenotypic transition of VSMCs, and they play important roles in the progression of VC and lead to cardiovascular disease [92,93]. The canonical Wnt signaling and its downstream target genes are required for the differentiation of osteoblasts. Gaur et al. reported that Wnt signaling activates the RUNX2 promoter and induces endogenous RUNX2 gene expression in osteoprogenitor cells. Furthermore, both Wnt and TCF1 can synergistically enhance RUNX2 promoter activity during osteoblastogenesis [94]. In fact, Wnt 3a, Wnt 5a, and Wnt 16 drive pro-osteogenic effects [95,96], while Wnt 1 has an anti-osteogenic effect on osteoblast progenitor cells [97]. 

Histone acetyltransferase p300 is a transcriptional coactivator of diverse transcription factors, and acetylation of β-catenin by p300 potentiates β-catenin signaling activation by several mechanisms [98,99]. In normal conditions, Sirt1 binds to p300 and inhibits β-catenin translocation into the nucleus, which prevents osteogenic activity and VC. However, a lower expression of Sirt1 in a high-glucose environment can no longer prevent the acetylation by p300 and phosphorylation by glycogen synthase kinase-3 beta (GSK-3β) of β-catenin, which subsequently facilitates β-catenin binding to TCF/LEF and driving RUNX2 and Bone morphogenetic protein (BMP) transcription (Figure 3, middle) [100]. 

BMPs, members of the TGF-β superfamily, are responsible for osteoblast differentiation and bone formation. BMP-2 is associated with phosphate-induced VC under high levels of phosphate but not in normal phosphate conditions, because BMP-2 promotes phosphate uptake in VSMCs in a dose-dependent manner by upregulating Pit-1 expression. Moreover, BMP-2 can directly induce RNX2 expression and suppress SM-22 expression, which implies that BMP-2 also participates in the phenotypic transition of VSMCs [101]. BMP-2 also has a synergistic effect on β-catenin to accelerate mineralized matrix deposition in VC [102]. High mobility group box 1 (HMGB1) is an activator of BMP-2, and it is involved in systemic inflammation. Deacetylation of HMGB1 by Sirt1 facilitates its nuclear-to-cytoplasmic translocation and prevents the inflammatory reactions that predisposes one to VC [103,104]. 

## 5. Role of Sirt1 and Sirt1 Modulator in Clinical Setting

### 5.1. Sirt1 Retard Hyperphosphatemia-Induced Medial Calcification in CKD

Several types of abnormalities increase the burden of phosphorus on the body and predisposes one to VC in vivo, for example, CKD in a high-phosphorus diet, Klotho or FGF-23 genetic defects that impair urinary phosphorus excretion, vitamin D intoxication, or parathyroid suppression. Numerous studies have shown high phosphate levels are capable of initiating or promoting matrix calcification in VSMCs [105], and this is mediated by the type III sodium-dependent phosphate cotransporter Pit-1 [106]. There are several mechanisms that have been proposed regarding VC induced under high phosphate levels, including VSMCs osteogenic conversion, VSMCs apoptosis, loss of inhibitor, and extracellular matrix (ECM) deposition [107]. High phosphate levels upregulate the gene expression of osteoblast/osteogenic phenotypes (RUNX2, osteocalcin, OPN, bone morphogenetic protein-2 (BMP-2), and ALP) [105,106,108] and simultaneously downregulate the gene expression of contractile phenotypes (SMαA and SM-22 α) [109,110]. In addition, calcium phosphate nanocrystals can also aggravate osteogenic phenotypic conversion by increasing expressions of BMP-2 and RUNX2 [111,112]. Growth arrest-specific gene 6 (Gas6), and its receptor Axl1, act as anti-apoptosis molecules and are involved in inhibiting osteogenic differentiation of vascular pericytes in response to vascular injury [113,114,115]. High phosphate levels downregulate Gas6 and Axl1 and contribute to VSMCs apoptotic cell death, consequently preventing release of the matrix vesicle to concentrate and crystalize calcium, which initiates VC [116]. Intriguingly, hydroxyapatite crystals also have the ability to induce VSMCs apoptotic cell death [117].

High phosphate levels also stimulate VSMCs cellular senescence in CKD animals by increasing β-galactosidase-positive cells, both in aortic medial areas with marked calcification and in cultured VSMCs [118]. Treatment of VSMCs with high phosphate levels in vitro can significantly decrease Sirt1 expression and consequently increase its acetylated form, such as histone-3 and p53. Sirt1 downregulation induced by high phosphate levels leads to osteoblast phenotypic switching of VSMCs and is attenuated by p21 knockdown. Combined treatment with high phosphate levels and phosphonoformic acid (PFA), an Na-dependent phosphate cotransporter inhibitor, can restore Sirt1 levels and reverse its deacetylated downstream targets [118]. Therefore, Sirt1 is involved in hyperphosphatemia-related VC development, and rescue of Sirt1 expression is reasonable to inhibit cell senescence and osteogenic phenotype switching of VSMCs. 

Hyperphosphatemia has been identified as an independent risk factor for cardiovascular disease in dialysis patients [119,120,121]. Even subtle increases of the phosphate level are associated with increased mortality in normal or early kidney disease patients [122,123]. Hence, treatment to lower phosphate burden is the cornerstone of therapy for hyperphosphatemia-related cardiovascular disease. Phosphate binders are designed to counteract dietary phosphorus overload in renal failure. In adenine-based diets in CKD animals, phosphate binders can restore CKD-induced hyperphosphatemia, decrease FGF-23 levels, and diminish urinary phosphate excretion, and they concurrently alleviate calcium and phosphate contents in the aorta. The gene expressions for osteoblastic phenotype transitions (ALP, RUNX2, p16, and p21) are ameliorated after treatment with phosphate binders in CKD mice [124]. The relationship between serum phosphate and risk of death is U-shaped in the hemodialysis population [125]. Nevertheless, there is still controversy regarding the protective role of phosphate binders on VC, and any clinical outcomes need larger-scale, randomized trials to be further investigated. In addition, there is no available study discussing the impact of phosphate reduction on survival, and the mechanism of phosphate binders on the expression of Sirt1 needs to be further explored. 

### 5.2. Vitamin D Supplement Is Beneficial in ECs and Adipose Tissue by Upregulation of Sirt1

Vitamin D deficiency (< 20 ng/mL) and insufficiency (20–29 ng/mL) are common among patients with CKD or who are undergoing dialysis. The benefit of vitamin D in mineral metabolism and skeletal health is well-documented in the literature. In the endothelium, vitamin D modulates macrophage and T lymphocyte activity, and it exerts anti-inflammatory and antibacterial activities [126,127]. Vitamin D supplements show extensive benefits if the population has vitamin D deficiency [128,129,130,131,132]. In CKD patients, uremic toxins contribute to the inflammatory response and lead to immune dysregulation. Higher levels of Toll-like receptor 4 (TLR4), cathelicidin, and MCP-1 are observed in uremic milieu, and they can be recovered after vitamin D supplements [133]. Our previous studies also demonstrated vitamin D supplements can significantly increase cathelicidin levels and additively reduce the parathyroid hormone in treating secondary hyperparathyroidism in patients undergoing maintenance hemodialysis [134,135]. Actually, vitamin D supplements in patients with vitamin D deficiency can restore the vitamin D levels and offers a protective role against VC. However, high doses (supraphysiological) of vitamin D given or vitamin D supplements in patient without vitamin D deficiency will be harmful. 

In ECs, vitamin D has been shown to have a protective role against oxidative stress-induced apoptosis [136]. Vitamin D treatments can reduce superoxide anion generation in ECs, and it can inhibit cell apoptosis through phosphorylation of the MEKs/ERKs-signaling pathway. Sirt1 is the downstream phosphorylation target of ERK, and it acts as a key regulator of vitamin D-mediated antioxidant properties in ECs [136]. In addition, vitamin D has the ability to inhibit EC senescence by activating the MEKs/ERKs-signaling pathway and upregulating Sirt1 expression [137]. In obese rats fed a diet deficient in vitamin D, the expressions of Sirt1 and AMPK in adipose tissue were significantly decreased, and PPARγ was upregulated to induce macrophage polarization and infiltration [138]. It is reasonable that an adequate level of vitamin D supplement is beneficial in attenuating macrophage-induced inflammation in adipose tissue [139]. Vitamin D supplements in adipose tissue treated with high levels of glucose led to enhanced phosphorylated AMPK/Sirt1 expression and antioxidant enzyme-related Nrf2 transcription factor, and both stimulated GLUT4 expression, which increased glucose uptake in adipose tissue. That is, vitamin D supplements can both abate oxidative stress and improve glucose metabolism in adipose tissue [140]. Besides, vitamin D supplements can restore mitochondrial dysfunction and decrease lipid accumulation caused by excess palmitic acid through the upregulation of Sirt1 and AMPK [141]. In sum, supplementing with vitamin D in vitamin D deficiency can attenuate EC oxidative stress and alleviate the inflammatory response in adipose tissue. 

### 5.3. Sirt1 Activator Modulates Vascular Disease in Preclinical and Clinical Settings

Sirt1 activators have been proposed as a therapeutic strategy for treating and preventing vascular disease. Several Sirt1 activators have been synthesized, such as SRT1720, SRT2014, and SRT1460, and these activate Sirt1 through an allosteric mechanism. SRT1720 would reduce superoxide production and alleviate inflammation in the aorta and thereby lower the aortic pulse wave velocity, which represents a large elastic artery stiffness [142]. SRT1720 reverses endothelial dysfunction in aged mice by enhancing COX-2 expression and reducing oxidative stress [143]. MHY2233 is another potent Sirt1 activator; it has been proposed to delay the aging process by decreasing senescence-associated β-galactosidase activity and cellular senescence biomarkers. MHY2233 can improve the proliferation, migration, and overall function of EPCs, which is a primary potential strategy in treating cardiovascular disease [144]. 

In type 2 diabetic patients, SRT1720 ameliorates atherosclerotic plaque formation and improves insulin resistance by repressing NF-κB signaling [145]. A pilot double-blind randomized, controlled study demonstrated SRT2104 is a safe and well-tolerated compound in the elderly. SRT2104 is proven to be beneficial in lowering the lipid profile and possibly increases insulin sensitivity in animal models with hyperlipidemia, diabetes, and obesity [146,147]. At present, more ongoing clinical trials are underway to investigate the efficacy, pharmacokinetics, and safety of Sirtuin modulator compounds in several diseases (http://clinicaltrials.gov). 

Resveratrol is a polyphenolic plant extract that possesses significant antioxidant and anti-inflammatory properties. It has been shown to greatly enhance Sirt1 transcription and activity [51]. Compared with synthetic Sirt1 activators, resveratrol is a natural compound and is less potent and more soluble and bioavailable due to the absence of a hydrogen bond and having no charged interaction residues [81]. Figure 3 illustrates the protective role of resveratrol in attenuating VC. In endothelium, FoxOs transcription factors are downstream signals of Sirt1, and they have been shown to be associated with resveratrol-induced eNOS expression [62]. Resveratrol induces Sirt1 and FoxO3a expression and then suppresses cellular ROS production under a hyperglycemic environment [63]. In PVAT, resveratrol can ameliorate adipokine release from dysregulated PVAT in response to inflammatory insult by AMPK/Sirt1 in an interdependent manner [89]. Resveratrol can also alleviate oxidative stress-induced cytokine release from PVAT and, subsequently, can improve arterial wall hypertrophy and adventitial collagen I accumulation, which results in arterial stiffness in aged mice [90]. In hyperphosphatemia-induced VC in CKD, resveratrol can significantly alleviate VSMC senescence and VC by activation of Sirt1. Additionally, resveratrol can abolish RUNX2 activation induced by high phosphate levels. Further, hyperphosphatemia induces VC by osteogenic conversion, apoptosis, and senescence of VSMCs through the Pit-1 cotransporter, which can be retarded by the Sirt1 activator resveratrol. Therefore, resveratrol has a protective role against medial calcification related to high phosphate levels through inhibiting cell senescence and osteogenic phenotype switching of VSMCs.

## 6. Conclusions

VC occurs in the intimal and medial layers of blood vessels and is highly associated with mortality. Deficiency of endothelial NO bioavailability, senescence of ECs and VSMCs, osteogenic phenotypic transdifferentiation, apoptosis of VSMCs, ECM deposition, and the inflammatory response of PVAT contribute to the development of VC. Numerous studies have shown Sirt1 can directly attenuate VC by reversing dysregulation of ECs, VSMCs, and PVAT. The inhibitory effect of Sirt1 on Wnt/β-catenin can further alleviate the VC process. Restoring Sirt1 levels by vitamin D supplements exert antioxidant and anti-inflammatory properties in ECs and adipose tissue to prevent VC. Sirt1 activators, such as resveratrol or the synthetic Sirt1 compound, have been proposed as therapeutic strategies for treating and preventing vascular calcification.

## Abbreviations

AMPK	Adenosine monophosphate-activated protein kinase
BMP-2	Bone morphogenetic protein-2
CR	Calorie restriction
CKD	Chronic kidney disease
DM	Diabetes mellitus
ECM	Extracellular matrix
FoxO	Forkhead box O
GSK-3β	Glycogen Synthase Kinase-3 beta
MGP	Matrix Gla protein
NAM	Nicotinamide
NO	Nitric oxide
NOS	NO synthase
PPAR	Peroxisome proliferator-activated receptor
PVAT	Perivascular adipose tissue
ROS	Reactive oxygen species
RUNX2	Runt-related transcription factor-2
SMαA	Smooth muscle α-actin
SM-22α	Smooth muscle-22α
SIPS	Stress-induced premature senescence
Sirt1	Sirtuin-1
VC	Vascular calcification
VSMCs	Vascular smooth muscle cells
